# The effect of obesity on metabolic parameters: a cross sectional study in adult women

**DOI:** 10.4314/ahs.v22i4.29

**Published:** 2022-12

**Authors:** Seyit Ramazan Karadogan, Eren Canbolat, Funda Pınar Cakıroglu

**Affiliations:** 1 Ankara University, Institute of Health Sciences, Department of Nutrition and Dietetics, Ankara, Turkey; 2 Ondokuz Mayıs University, Faculty of Tourism, Department of Gastronomy and Culinary Arts. Samsun, Turkey; 3 Ankara University, Faculty of Health Sciences, Department of Nutrition and Dietetics, Ankara, Turkey

**Keywords:** Obesity, Abdominal Fat, Blood Chemical Analysis, Women's Health, Anthropometry

## Abstract

**Background:**

In this study conducted in adult healthy women, it was aimed to determine the relationship between anthropometric measurements such as Body Mass Index (BMI), waist circumference, waist/height ratio and blood parameters, which are used to define obesity.

**Methods:**

A total of 90 women, with a mean age of 38.4±8.8 years, 34 pre-obese between BMI: 25.0–29.9 kg/m2 and 56 obese BMI: ≥30.0 kg/m2, participated in the study.

**Results:**

According to plasma atherogenic index (PAI), women with high cardiovascular risk had lower high-density lipoprotein (HDL) values, while low-density lipoprotein (LDL), triglyceride (TG), aspartate transaminase (AST) values were found to be higher (p<0.05). It was observed that obese women according to BMI had higher fasting glucose, TG, alanine transaminase (ALT), AST and urea values, and lower HDL and mean erythrocyte volume (MCV) values compared to pre-obese women (p<0.05). A moderately positive relationship was found between body fat percentage, waist circumference and waist/height ratio and fasting glucose and ALT levels of women, and a moderate negative relationship was found between vitamin D levels (p<0.05).

**Conclusions:**

It is understood that the increase in body fat tissue, especially in the abdominal region, in adult healthy women has negative effects on blood parameters and the risk of developing chronic diseases will decrease by preventing this situation.

## Introduction

It is thought that obesity, which is defined as abnormal or excessive fat accumulation that may impair health in the body, is caused by the imbalance between energy intake and expenditure^1^,^2^, however, studies conducted in recent years have also associated obesity with various factors such as appetite mechanism^3^, microbiota^4^, endocrine disruptors, epigenetics^5^, circadian rhythm^6^, sleep duration^7^ and activation of 5′-adenosine monophosphate-activated protein kinase (AMPK)^8^. According to the latest data, in addition to island countries such as Nauru, Palau and Niue, which are known to have more than half of the world's populations obese, Kuwait (37.9%), the United States (36.2%), Saudi Arabia (35.4%), Qatar (35.1%), Lebanon (33.7) Libya (32.5%) and Turkey (32.1%) are the countries where obesity is most common in the world^9^. Obesity, the prevalence of which is increasing worldwide, causes metabolic and endocrine changes, leading to an increase in chronic diseases such as cardiovascular diseases, diabetes, respiratory system diseases, osteoarthritis, cancer^10^,^11^.

While there is more than one method to determine obesity, in practice, the definition and classification of obesity is made with the Body Mass Index (BMI), which is obtained by dividing the weight in kilograms of adults by the square of the height in meters^12^. In adult individuals, those with a BMI between 25.0–29.9 kg/m^2^ are defined as overweight or pre-obese, and those with a BMI above 30.0 kg/m^2^ are defined as obese 13. However, it is known that BMI is related to the total body mass and does not provide clear information about the distribution of fat and the type of obesity^14^, and it is stated that the amount of body fat is a better indicator for defining obesity, rather than BMI^15^. While the ideal body fat ratio is 25% in women and 15% in men16, a body fat ratio of >35% in women and >25% in men is considered as obesity^15^.

Today, in addition to the total amount of fat in the body, obesity definitions are made according to the region and distribution of fat in the body. The distribution of fat throughout the body is defined as ovoid type, and the accumulation under the skin around the waist and abdomen in the upper part of the body is defined as central type (visceral/abdominal) obesity^17^. While waist circumference, waist/hip ratio and waist/height ratio are used to determine central obesity^16^, waist/height ratio seems to be superior to both BMI and waist circumference measurements^18^. Central obesity leads to metabolic syndrome, which is defined as a fatal endocrinopathy in which systemic disorders such as insulin resistance, glucose intolerance, diabetes, dyslipidemia, hypertension, and coronary artery disease are combined^19^–^21^.

It is a known fact that obesity increases the risk of chronic diseases by causing metabolic damage. In this study, which was carried out to reveal this situation, the effects of anthropometric measurements used in the definition of obesity on some blood parameters were examined in pre-obese and obese women who have not yet been diagnosed with chronic diseases. Thanks to the results of the study, it will be understood more clearly what kind of effects obesity has on metabolic parameters and the risk of developing chronic diseases in healthy individuals. This will enable the obesity factor to be taken into account in healthy and sick individuals. At the same time, an awareness for preventive medicine will be created and the necessity of considering obese individuals who have not yet suffered from chronic diseases will be revealed.

## Material and Method

### Study Population

Female individuals between the ages of 20–50 (mean 38.4±8.8 years) with BMI>25.0 kg/m2, who voluntarily applied to the Central Community Health Center Obesity Unit affiliated to the Zonguldak Provincial Health Directorate October 2017 and March 2018, participated in this observational, analytical and cross-sectional study. In the study, individuals with chronic diseases (such as endocrinological diseases, cardiovascular diseases, diabetes, cancer, psychiatric diseases), individuals using drugs (levothyroxine, anti-thyroid, anti-hypertensive, oral or parenteral diabetes drugs, lipid-lowering drugs, antidepressants, steroids, oral contraceptives and psychiatric drugs), pregnant women, those using nutritional support, and women who did not have a general screening and blood chemical analysis at the Community Health Center at least 1 month ago were not included. Following the exclusion criteria, the study was completed with 90 pre-obese and obese female individuals. It is seen that the majority of women are married (73.3%) and have applied to a nutritionist at least once (64.4%) for weight loss. Various findings of the participants are given in Table 1. Approval for the research was acquired from the Ethics Board of Ankara University (15.09.2017-15/237). Each participant in the research signed a consent form prior to his or her inclusion in the study.

**Table 1:** Various findings of participants

Variable		n	%
Education Status	Primary Education Degree	36	40.0
	High School Degree	32	35.6
	Bachelor's degree	22	24.4
Married Status	Single	24	26.7
	Married	66	73.3
Diet History	Yes	58	64.4
	No	32	35.6
Regular Physical Activity	Yes	23	25.6
(150 minute/week)	No	67	74.4

### Anthropometric Assessment

The height of the women participating in the study was measured when the feet were together and the head was in the Frankfort plane, and a stadiometer was used in the measurement. According to BMI values, 25.0–29.9 kg/m^2^ was considered as pre-obese and ≥30.0 kg/m^2^ was considered as obese. Waist circumference was measured at the midpoint between the lowest rib on the right side and the hip bone prominence (iliac) while the person was standing. Waist/height ratio was calculated by dividing waist circumference by height. Central obesity is defined as a waist circumference of 100 cm in men, 90 cm in women and a waist/height ratio above 0.521-23.

### Body Composition Analysis

The body compositions of the participants (body weight, body fat percentage, body fluid percentage, muscle mass) were measured with a single frequency (50 kHz) bioelectrical impedance analysis method, and the Tanita SC 330 Body Analysis device was used for this measurement. The measurement was taken after the participants were informed about the measurement rules one day before. These rules are that participants should not do heavy physical activity 24–48 hours before the measurement, not drink alcohol 24 hours before, eat at least 2 hours before, not drink much water before the test, not drink tea and coffee 4 hours before the test, and not have metal jewellery and a pacemaker on the individual.

### Blood and Other Analysis

The blood parameters of the women were determined by examining the patient registry files. In this context, the results of fasting blood sugar, total cholesterol, high-density lipoprotein (HDL), low-density lipoprotein (LDL) cholesterol, triglyceride (TG), aspartate transaminase (AST), alanine transaminase (ALT), creatinine, urea, hemoglobin (HB), hematocrit (HCT), mean red cell volume (MCV), vitamins B12 and D were obtained from the analyzes made in the files within the last month. The plasma atherogenic index (PAI), obtained as the base-10 logarithm of the ratio of plasma TG to HDL [log (TG/HDL-C)], was used to assess participants' cardiovascular risk. Individuals were classified as low risk (<0.11), intermediate risk (0.11–0.24) and high risk (>0.24) according to PAI value^25^.

### Statistical Analysis

The analysis of the data was performed using the SPSS 21.0 (SPSS Inc, Chicago, IL, USA) software program. Research data are presented in tables with absolute (n) and percent (%) values. Means and standard deviation (X±S) values were taken if there are necessary. The presence of a normal data distribution was evaluated with the Kolmogorov-Smirnov test. In cases where the assumption of normality was met, the Independent Samples t-test was used to compare two independent groups, and the One-Way Anova test was used to compare more than two groups. To determine the differences between more than two groups, Bonferroni or Tamhane post hoc tests were applied according to the Levene's Test results. In cases where the assumption of normality was not met, the Mann Whitney-U test was used to compare two independent groups, and the Kruskal Wallis-H test was used to compare more than two groups. The relationship between two quantitative variables was determined by Partial Correlation Analysis by taking control of one or more variables that are likely to affect these variables. According to the correlation coefficient between the variables 0<r≤0.3=weak; 0.3<r≤0.7=medium; 0.7<r≤1.0=strong correlation evaluation was made. The statistical significance level was p<0.05 and the confidence interval was 95% 26,27.

## Results

The results of the participants' body composition, anthropometric measurements and PAI values are given in Table 2. According to BMI values, 37.8% of women were found to be pre-obese and 62.2% to be obese. It is seen that central obesity occurs in 92.2% of women according to waist circumference measurements and in 97.8% according to waist/height ratios. Finally, it was determined that 12.2% of the participants had a moderate risk for cardiovascular diseases , and 68.9% had a high risk.

**Table 2:** Measurements and groups of participants

Variable		X ± S	Min-Max
Height (cm)		158.8±5.8	140.0–171.0
Weight (kg)		82.3±14.2	58.0–122.4
Fat Percentage (%)		39.1±5.3	26.6–51.2
Fluid Percentage (%)		43.4±3.3	36.0–50.9
Muscle Mass (kg)		47.0±5.2	38.9–69.3
BMI (kg/m2)		32.7±5.9	25.1–50.7
Waist Circumference (cm)		105.0±11.5	83.0–135.0
Waist/Height Ratio		0.66±0.08	0.49–0.91
PAI		0.35±0.26	−.20–1.10
		n	%
BMI (kg/m2)	25.0–29.9	34	37.8
	≥30	56	62.2
Waist Circumference (cm)	<90	7	7.8
	≥90	83	92.2
Waist/Height Ratio	<0.5	2	2.2
	≥ 0.5	88	97.8
PAI	<0.11	17	18.9
	0.11–0.24	11	12.2
	>0.24	62	68.9

The relationship between the blood parameters of the women and their PAI, BMI and physical activity status is shown in Table 3. According to PAI, women with high cardiovascular risk had lower HDL values, while LDL, TG, and AST values were higher (p<0.05). According to BMI, obese women had higher fasting glucose, TG, ALT, AST and urea values than pre-obese women, and lower HDL and MCV values (p<0.05). In addition, it was determined that the LDL and TG values of women who do regular physical activity are lower than those who do not (p<0.05).

**Table 3:** Relationship of various findings with blood parameters

Variable		Fasting Glucose mg/dL	Total Cholesterol mg/dL	HDL mg/dL	LDL mg/dL	TG mg/dL	ALT U/L	AST U/L
X ± S (All participants)		99.2±21.3	192.5±42.9	51.2±12.4	115.8±36.9	125.1±66.2	17.9±8.5	18.8±5.7
PAI	<0.11	90.4±10.2	168.0	65.7±15.5a	102.0	62.1±17.1	16.0±7.1	19.0
	0.11–0.24	96.1±19.1	156.0b	50.6±8.6	103.0	76.1±14.1b	13.7±3.8	16.0b
	>0.24	102.2±23.3	193.0	47.3±8.7c	116.0c	151.1±63.7c	19.1±9.2	19.0
BMI (kg/m2)	25.0–29.9	93.3±19.4*	181.0	55.7±15.4*	108.8±31.5	89.0*	14.8±5.6*	17.1±3.8*
	≥30	102.8±21.8*	187.0	48.5±9.3*	120.0±39.4	125.0*	19.7±9.5*	19.8±6.4*
Regular Physical Activity (150 minute/week)	Yes	96.0	173.0	52.0	105.6±29.4*	90.0*	14.0	18.0
	No	95.0	193.0	48.0	119.2±38.7*	123.0*	17.0	22.0

Variable		Creatinine mg/dL	Urea mg/dL	HB (g/dL)	HCT %	MCV (fL)	Vit B12 pg/mL	Vit D ng/mL
X ± S (All participants)		0.73±0.12	22.8±6.8	12.5±1.2	39.7±3.1	85.5±5.2	339.8±177.3	19.4±15.3
PAI	<0.11	0.7	22.3±5.9	12.3	39.0±3.4	88.5	321.0	14.7
	0.11–0.24	0.7	22.7±6.0	12.1	39.3±2.5	87.6	308.5	8.9
	>0.24	0.7	23,3±7.3	12.5	40.0±3.2	85.8	298.0	16.7
BMI (kg/m2)	25.0–29.9	0.7	21.1±5.8*	12.6±1.2	39.6	87.9*	309.0	15.6
	≥30	0.7	23.9±7.2*	12.4±1.2	39.5	85.1*	280.5	15.1
Regular Physical Activity (150 minute/week)	Yes	0.7	22.4±5.7	12.4±1.1	39.6±2.5	86.3	278.0	16.8
	No	0.7	23.0±7.2	12.5±1.3	39.8±3.4	85,9	310.0	14.4

Data are given as mean±standard deviation for parametric variables and median for non-parametric variables. p<0.05 (a. <0.11 / 0.11–0.24 b. 0.11–0.24 / >0.24 c. <0.11 / >0.24)

* p<0.05

**Figure 1:**
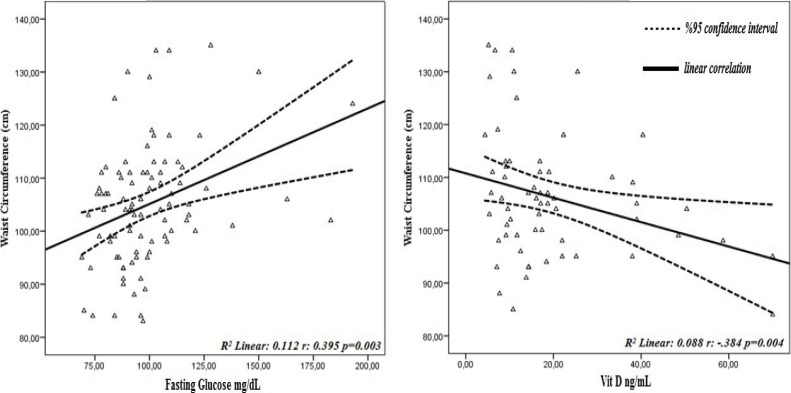

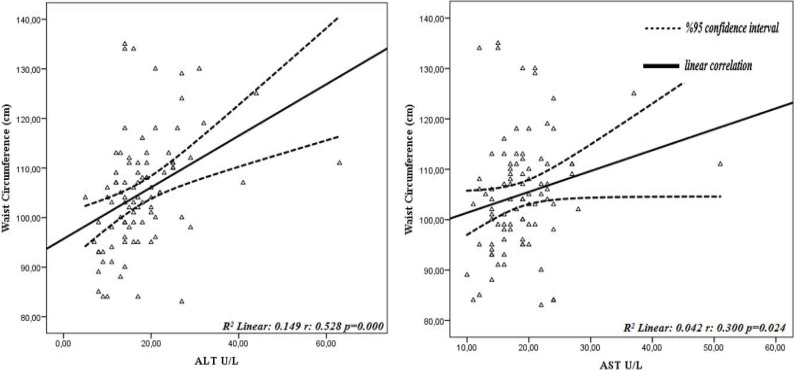
Effect of waist circumference on blood parameters

**Figure 2:**
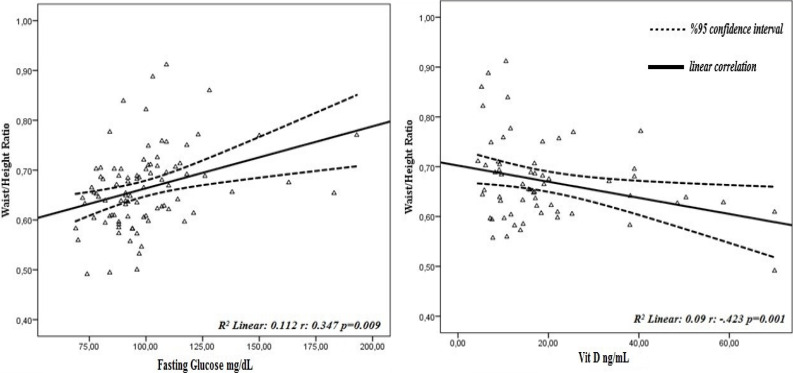

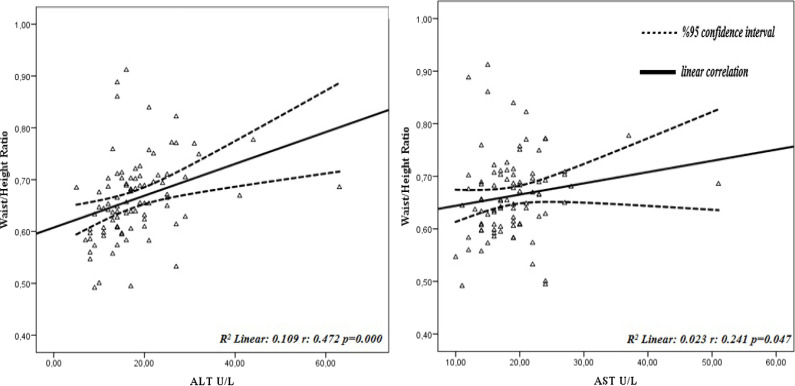
Effect of waist/height ratio on blood parameters

The relationship between blood parameters according to body fat percentage, waist circumference and waist/height ratio, which gives more accurate results than BMI in defining obesity, is given in Table 4. A moderately positive relationship was found between body fat percentage, waist circumference and waist/height ratio and fasting glucose and ALT levels of women, and a moderate negative relationship was found between vitamin D levels (p<0.05). As the body fat percentage and waist circumference and waist/height ratio of the women increased, a weakly level positive and significant relationship was observed for AST levels. (p<0.05). The effects of waist circumference and waist/height ratio, which are central obesity markers, on blood parameters are shown graphically in Figure 1 and 2.

**Table 4:** The relationship between obesity indicators and blood parameters

Variable	Fasting Glucose mg/dL	Total Cholesterol mg/dL	HDL mg/dL	LDL mg/dL	TG mg/dL	ALT U/L	AST U/L
Fat Percentage (%)	.386*	-0.27	-.173	.033	.046	.477*	.282*
Waist Circumference (cm)	.395*	-.055	-.183	.018	-.013	.528*	.300*
Waist/Height Ratio	.347*	-0.33	-.194	.047	.024	.471*	.241*

Variable	Creatinine mg/dL	Urea mg/dL	HB (g/dL)	HCT %	MCV (fL)	Vit B12 pg/mL	Vit D ng/mL
Fat Percentage (%)	.073	.036	.004	.053	.-145	.-192	.-312*
Waist Circumference (cm)	-.097	-.032	.003	.065	-.101	-.166	-.384*
Waist/Height Ratio	-.090	.-044	.011	.088	.-100	.-187	.-423*

The age variable was controlled.

* p<0.05

## Discussion

Obesity, which has increased three times in the last 50 years and has become a global pandemic affecting more than 650 million people^2^ and associated with an increase in body fat, especially in the abdominal region, increases the morbidity risk of cardiovascular diseases, diabetes, metabolic syndrome and some types of cancer^28^. Inflammation, which becomes chronic with the secretion of inflammatory cytokines such as tumour necrosis factor-α (TNF-α) and interleukin-6 (IL-6) in excess in the adipose tissue that increases in obesity, causes pathological changes in insulin-sensitive tissues and β-cells, leading to disruption of metabolic regulation^29^. In addition, due to decreased angiogenesis, increased vasoconstriction and decreased blood flow to the adipose tissue in obese individuals, the oxygen levels of the adipose tissue also decrease, and accordingly, fat accumulation and systemic immune problems are seen together with the disorders occurring in the adipose tissue^30^.

This study was conducted to examine the effects of obesity status on blood parameters of adult women who do not have any chronic diseases, with the thought of predicting possible adverse events that may occur with obesity. The women participating in the study were pre-obese (37.8%) and obese (62.2%), and almost all of them were found to have central obesity. The fact that women have a high cardiovascular risk (68.9%) according to their PAI values confirms that central obesity is an important risk factor for cardiovascular diseases (Table 2). In a similar study, it was determined that 76.9% of obese individuals had a high cardiovascular risk according to their PAI values^15^. There are many studies in the literature that obesity increases the risk of cardiovascular disease^31^–^33^.

When the mean blood parameters of the participants are examined, it is seen that fasting glucose:99.2±21.3 mg/dL (reference value: <100 mg/dL), total cholesterol: 192.5±42.9 mg/dL (reference value: 0–200 mg/dL), HDL:51.2±12.4 mg/dL (reference value: 40–60 mg/dL), LDL:115.8±36.9 mg/dL (reference value: <130 mg/dL), TGA:125.166.2 mg/dL (reference value: 35–150 mg/dL), ALT:17.9±8.5 U/L (reference value: 0–34 U/L), AST:18.8±5.7 U/L (reference value: 0–31 U/L), creatinine:0.73±0.12 mg/dL (reference value: 0,66–1,09 mg/dL), urea:22.8±6.8 mg/dL (reference value: 10–40 mg/dL) HB:12.5±1.2 g/dL (reference value: 12–16 g/dL), HCT: 39.7±3.1% (reference value: 37–47%) MCV:85.5±5.2 fL (reference value: 87±7 fL), vitamin B12:339.8±177.3 pg/mL (reference value: 250–1100 pg/mL) vitamin D:19.4±15.3 ng/mL (reference value: 16–42 ng/mL).

At this point, it was determined that the mean blood parameters of the participants were within normal limits. However, in the statistical analysis, it was determined that as the PAI values of women increased, HDL values decreased and LDL, TGA, AST values increased, and as BMI values increased, fasting glucose, TGA, ALT, AST and urea values increased and HDL, MCV values decreased (p<0.05). In addition, LDL and TGA values of women who stated that they exercised at least 150 minutes a week were found to be significantly lower (p<0.05) (Table 3). There are studies in the literature in which obesity has negative effects on blood parameters^15^,^34^–^38^ and exercise have positive effects in this direction^39^–^41^.

In the partial correlation analysis performed by controlling the effect of the age variable on the blood findings, a moderate positive correlation was found with the body fat percentage, waist circumference and waist/height ratio, fasting glucose and ALT values of the women, and a weak positive correlation with the AST values (p<0.05). These results suggest that central obesity may result in impaired fasting glucose and liver damage. Another remarkable finding is that as the body fat percentage, waist circumference and waist/height ratio of women increase, vitamin D levels decrease significantly (p<0.05) (Table 4). In obese individuals, serum levels decrease with increased storage of vitamin D in adipose tissue and decreased production in the skin; In this case, it is stated that it increases adiposity by stimulating lipogenesis and providing calcium influx in adipocyte^42^. Similarly, there are studies in the literature that show a negative relationship between obesity and vitamin D^43^,^44^. Finally, the effect of central obesity on fasting blood glucose, vitamin D and liver enzymes (ALT-AST) is shown in Figure 1 and Figure 2.

In this study, it was determined that central obesity affects blood findings negatively in adult pre-obese and obese women who have not yet been diagnosed with a chronic disease. This situation reveals the relationship between obesity and many chronic diseases such as type 2 diabetes, hypertension, cardiovascular diseases and cancer, and shows that the incidence of these diseases will decrease with the prevention of obesity. Our study is important in terms of taking steps to prevent obesity by drawing attention to this issue. The fact that obese individuals generally have chronic diseases in similar studies on this subject will also enable this study to be distinguished in the literature. Because our study was carried out in healthy pre-obese and obese individuals. Among the results of the study, the positive effect of exercise on blood lipids shows that exercise will be a good option for the prevention of cardiovascular diseases in obese individuals. In addition to all these, the positive effect of exercise on blood lipids among the results of the study shows that exercise will be a good option for the prevention of cardiovascular diseases in obese individuals.

## Limitations of Study

As a result of the study, although the effects of obesity in adult women on blood parameters were determined in accordance with the literature, the study has some limitations. First of all, the exercise habits of the participants were taken based on the statement, and the blood findings were obtained from the patient registry files. Another limitation is that the sleep quality and nutritional habits of the participants were not determined and food consumption records were not taken. In addition, male individuals were not included in the study because the majority of individuals who applied for obesity treatment were women. Finally, the fact that the study was conducted on individuals who did not have a chronic disease and whose blood chemical analysis and general screening were performed in the last month led to a relatively low sample size. In future studies that will examine the effects of obesity on blood parameters, taking into account physical activity, nutritional habits, sleep quality, daily energy and nutritional levels for both sexes and performing them in larger samples may provide clearer results.

## Conclusion

In this study, it was aimed to examine the relationships between anthropometric measurements of adult healthy pre-obese and obese women and some blood parameters. Although the blood findings of the women participating in the study were within the normal range, it was determined that the increased body fat had a negative effect on the blood findings. Especially, the negative effects of central obesity on fasting blood sugar, liver enzymes (ALT-AST) and vitamin D were clearly seen. In this case, it is obvious that by preventing obesity, the formation of many chronic diseases will be prevented. Thus, health expenditures for chronic diseases due to obesity will decrease and national economies will be positively affected. As a result, it is necessary to expand the treatment methods (exercise, psychological, pharmacological and medical nutrition therapy) applied to prevent obesity and to apply them under the regular control of health professionals in obese individuals.

## Abbreviations

AMPK: 5′-adenosine monophosphate-activated protein kinase
AST: Aspartate Transaminase
BMI: Body Mass Index
HB: Hemoglobin
HCT: Hematocrit
HDL: High-Density Lipoprotein
LDL: IL-6: Interleukin-6 Low-Density Lipoprotein
MCV: Mean Red Cell Volume
PAI: Plasma Atherogenic Index
TNF-α: Tumor Necrosis Factor-α
TG: Triglyceride
